# Molecular subtyping and prognostic risk characterization of head and neck squamous cell carcinoma based on lysosome-related genes

**DOI:** 10.1097/MD.0000000000034275

**Published:** 2023-07-14

**Authors:** Aichun Zhang, Yangzi Jin, Xinbo Zou, Shuo Zhang

**Affiliations:** a Department of Otolaryngology, The First Affiliated Hospital of Zhejiang Chinese Medical University (Zhejiang Provincial Hospital of Traditional Chinese Medicine), Hangzhou, Zhejiang, China; b Department of Breast Surgery, The First Affiliated Hospital of Zhejiang Chinese Medical University (Zhejiang Provincial Hospital of Traditional Chinese Medicine), Hangzhou, Zhejiang, China.

**Keywords:** consensus clustering, head and neck squamous cell carcinoma, lysosome, nomogram, prognosis

## Abstract

Lysosomes possess a multitude of biological functions and are known to play a crucial role in the proliferation and metastasis of head and neck squamous cell carcinoma (HNSCC). This study aims to systematically investigate the potential role of lysosomes-related genes (LRGs) in the development of heterogeneity and prognosis in HNSCC. Publicly available transcriptome and clinical data of HNSCC were obtained and analyzed using consensus clustering to identify molecular subtypes. A risk model based on LRGs was developed and evaluated, including its correlation with clinical features, immune infiltration, drug sensitivity, and response to immune therapy. Gene set enrichment analysis was conducted to explore relevant pathways, and a prognostic nomogram model for HNSCC was constructed and evaluated. In this study, we identified 542 LRGs that exhibited differential expression in HNSCC, with 116 of these being significantly associated with overall survival. Two LRGs-derived molecular subtypes were identified, which displayed significant differences in prognosis and immune cell infiltration. Additionally, a prognostic risk model was developed, which included 13 LRGs. This model successfully divided HNSCC into low-risk and high-risk groups with different prognoses and immune cell infiltrations. The LRGs-derived risk signature was associated with immune infiltration, clinical features, drug sensitivity and immunotherapy response. The good prognosis of the low-risk group was linked to the activation of immune response-related processes and the inhibition of pathways such as necroptosis and neutrophil extracellular trap formation. Patients in the low-risk group had better immune therapy response, while those in the high-risk group had higher drug sensitivity. Finally, our nomogram, which combines clinical N staging and LRG-derived model, demonstrated excellent prognostic evaluation performance as shown by decision curve analysis and calibration curve. The study provides a comprehensive analysis of the expression and prognostic significance of LRGs in HNSCC, leading to the identification of 2 distinct molecular subtypes and the development of a risk model based on LRGs.

## 1. Introduction

Head and neck cancer is a highly malignant tumor that affects approximately 600,000 people worldwide annually.^[1,2]^ This type of cancer can originate from various regions of the head and neck, including the oral cavity, nasopharynx, oropharynx, hypopharynx, larynx, and neck.^[1]^ The most prevalent form of malignant tumor in this region is the head and neck squamous cell carcinoma (HNSCC).^[3]^ Treatment options for HNSCC include surgery, radiotherapy, chemotherapy, and targeted immunotherapy.^[3]^ However, due to the lack of early detection and the asymptomatic nature of HNSCC, the 5-year survival rate for patients remains low at 40% to 50%.^[4,5]^ The absence of accurate early cancer diagnostic biomarkers and ideal preclinical models has hindered effective clinical management of HNSCC.^[6]^ Therefore, it is essential to explore new and effective biomarkers for treatment response evaluation, drug sensitivity analysis, and prognosis prediction.

The lysosome is a fundamental organelle responsible for the degradation and digestion of cellular waste and harmful substances. It plays a role in diverse cellular processes, including cell death, immune response, energy metabolism, cell signaling, and receptor recycling.^[7,8]^ Dysfunctional lysosomal function has been linked to the occurrence and progression of cancer,^[9,10]^ promoting cancer progression and metastasis by activating the AKT signaling pathway.^[11]^ Studies has shown that inhibiting lysosomal function can increase the sensitivity of cancer cells to chemotherapy.^[12]^ Moreover, inhibitors of lysosomal function can significantly increase the death of HNSCC cell lines induced by c6-ceramide nanoliposomes.^[13]^ Although lysosomal-related genes (LRGs) have been identified as promising biomarkers for HNSCC,^[14,15]^ these studies have been limited to individual genes. There is a lack of systematic research on the expression and prognostic value of LRGs in HNSCC.

**Figure 1.**
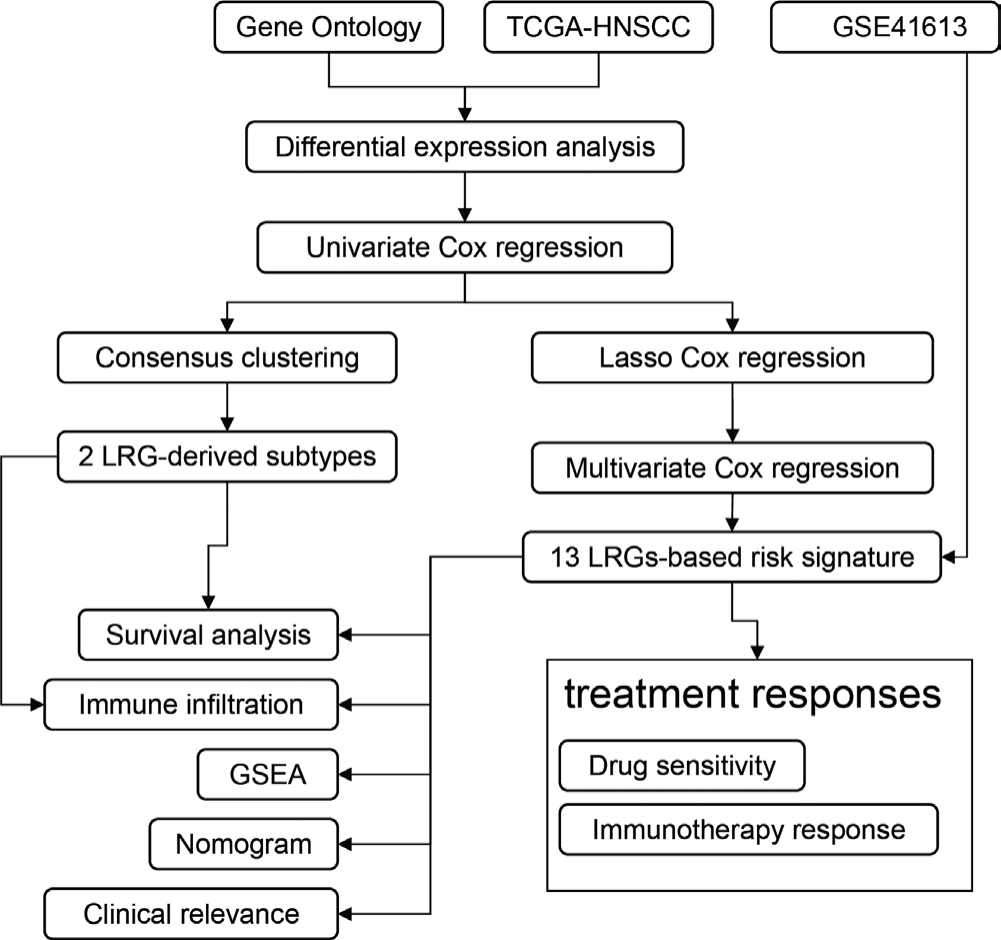
Flowchart of the development of HNSCC molecular subtyping and prognostic model based on LRGs. HNSCC = head and neck squamous cell carcinoma, LRGs = lysosomes-related genes.

This study aimed to utilize bioinformatics techniques to investigate the differential expression and prognostic significance of LRGs in HNSCC, identify molecular subtypes of HNSCC based on LRGs, and develop a risk model derived from LRGs for evaluating HNSCC prognosis, response to immune therapy, and sensitivity to chemotherapy. Figure 1 depicts the methodology employed in this research.

## 2. Materials and methods

### 2.1. Transcriptome and clinical data download for HNSCC

The transcriptome data and corresponding clinical data of the TCGA-HNSCC cohort were downloaded from the TCGA database, and cases with incomplete clinical information and prognosis data were excluded, resulting in a total of 439 HNSCC patients. Additionally, the validation set GSE41613 data was downloaded from the GEO database, which included 97 HNSCC patients. LRGs were obtained from the Gene Ontology website (http://geneontology.org/), resulting in a total of 875 outputs.

### 2.2. Molecular subtyping of HNSCC

The ConsensusClusterPlus R package was utilized to perform molecular subtyping on the TCGA-HNSCC cohort using prognosis-related differentially expressed LRGs transcriptome data. The limma package was first used to screen for differentially expressed LRGs in HNSCC (adjusted *P* value < .05 and |log(fold change)| > 1), and prognosis-related differentially expressed LRGs were identified through univariate Cox regression analysis (*P* value < .05).

### 2.3. Construction and evaluation of risk features

Lasso Cox regression analysis was utilized to compress prognosis-related differentially expressed LRGs, and multivariate Cox regression analysis was performed on the compressed genes to screen for independent prognosis-related differentially expressed LRGs with *P* value < .05. The risk score was then calculated using the following formula: riskscore= ∑(Gi*coefi) (“i” represents the number of genes, “Gi” represents the expression of each deLRGs, “coefi” represents the coefficient of each LRGs). The HNSCC cohort was divided into high-risk and low-risk groups using the median value, and the prognostic differences between the groups were evaluated using survival analysis. The prognostic performance of the risk model was also evaluated using receiver operating characteristic curve analysis.

### 2.4. Analysis of immune infiltration, drug sensitivity, and immune therapy response

The CIBERSORT R package was utilized to determine the immune cell composition of each patient in the TCGA-HNSCC cohort. The differences in immune infiltration between various molecular subtypes and high-risk/low-risk groups were compared. The sensitivity of each patient in the TCGA-HNSCC cohort to 6 commonly used chemotherapy drugs was analyzed using the pRRophetic R package. The immune therapy response was evaluated using Tumor Immune Dysfunction and Exclusion (TIDE) and Immunophenoscore (IPS). Standardized transcriptome data was submitted to the TIDE website (http://tide.dfci.harvard.edu) to calculate TIDE, Cancer-associated fibroblasts, Tumor-associated macrophages of M2 phenotyp, and other scores. The calculation of IPS was obtained by submitting the TCGA database labels of high-risk and low-risk group patients to The Cancer Immunome Atlas.

### 2.5. Gene set enrichment analysis

To investigate the differences in signaling pathways between high-risk and low-risk groups, we performed enrichment analysis of biological functions and KEGG pathways using the “clusterProfiler” package in R, and generated a bubble chart to display the results.

### 2.6. Construction and evaluation of nomogram

Univariate and multivariate Cox regression analyses were conducted to identify prognostic factors for HNSCC. A nomogram model was constructed using the rms R package, and its performance in predicting 1, 3, and 5-year overall survival of HNSCC patients was evaluated using calibration curves. Decision curves were utilized to compare the net benefit of the nomogram and other prognostic factors in predicting 1-year overall survival of HNSCC patients.

### 2.7. Statistical analysis

Data analysis and visualization were performed using R version 4.2.2 and relevant R packages. The Wilcoxon test was utilized for comparing intergroup differences. Univariate and multivariate Cox regression analyses were performed using the “survival” R package. Kaplan–Meier survival curves and log-rank test were employed for survival analysis. Statistical significance was defined as *P* value < .05.

## 3. Results

### 3.1. Molecular subtyping and prognosis analysis of HNSCC based on LRGs

**Figure 2.**
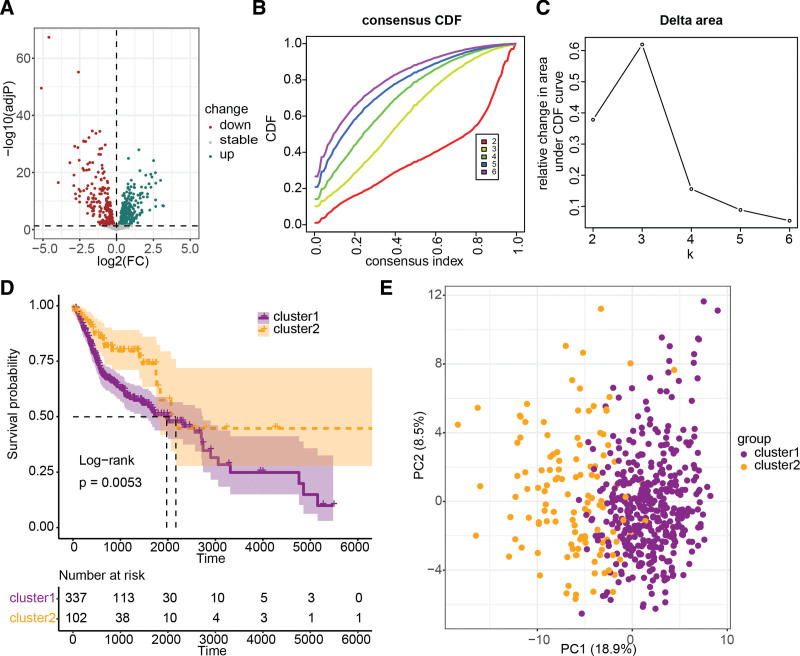
HNSCC molecular subtyping and prognostic evaluation based on LRGs. (A) Volcano plot of differentially expressed LRGs in HNSCC. (B and C) Consensus clustering of HNSCC based on prognostic-related LRGs. (D) Survival analysis of molecular subtypes derived from LRGs using K-M curves. (E) Principal component analysis of HNSCC based on prognostic-related LRGs. HNSCC = head and neck squamous cell carcinoma, LRGs = lysosomes-related genes.

The analysis of differential expression indicated that 542 LRGs exhibited differential expression in HNSCC, with 291 being upregulated and 251 being downregulated (Fig. 2A). Univariate Cox regression analysis identified 116 of these differentially expressed LRGs as being associated with overall survival in HNSCC (Supplementary Table S1, http://links.lww.com/MD/J248). Consequently, consensus clustering analysis was performed on these genes (Fig. 2B and C), which revealed 2 HNSCC molecular subtypes (cluster1 and cluster2). The Kaplan–Meier survival curves demonstrated a significant difference in prognosis between these 2 molecular subtypes, with cluster2 exhibiting a significantly better prognosis than cluster1 (Fig. 2D). Principal component analysis based on these prognostic-related LRGs revealed a clear boundary between cluster1 and cluster2 (Fig. 2E). These findings suggest a high degree of consistency between cluster1 and cluster2.

### 3.2. Construction and evaluation of prognostic signature derived from LRGs

**Figure 3.**
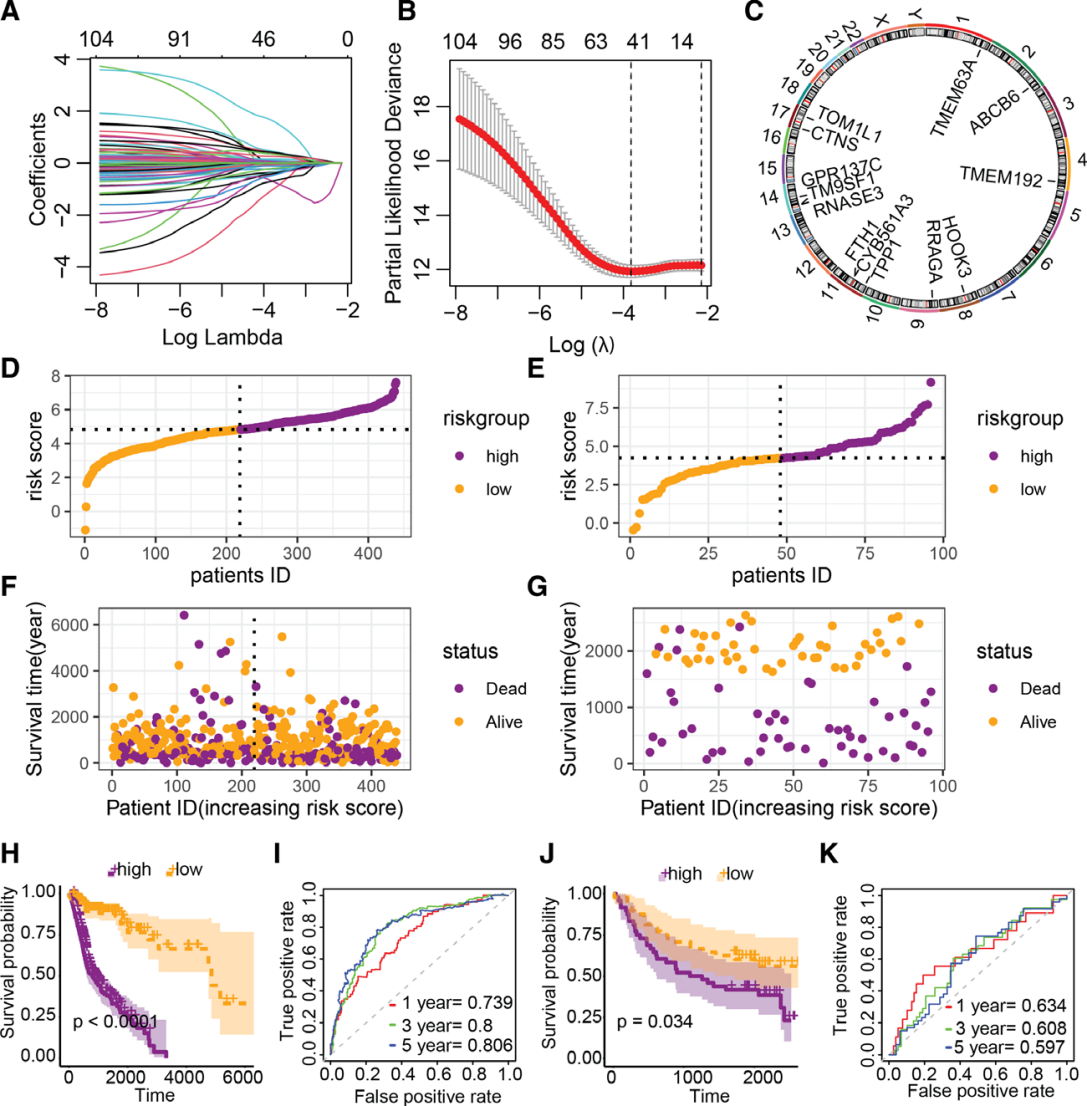
Construction and evaluation of HNSCC prognostic risk features derived from LRGs. (A and B) Lasso Cox regression analysis to reduce prognostic-related LRGs. (C) Chromosomal localization of LRGs included in multivariate Cox regression analysis. (D–G) Distribution of high-risk and low-risk groups based on risk features derived from LRGs in TCGA-HNSCC and GSE41613 cohorts. (H) K-M curves of survival analysis for high-risk and low-risk groups in TCGA-HNSCC cohort. (I) ROC curves and AUC values of the risk model for predicting 1, 3, and 5-yr overall survival in TCGA-HNSCC cohort. (J) K-M curves of survival analysis for high-risk and low-risk groups in GSE41613 cohort. (K) ROC curves and AUC values of the risk model for predicting 1-, 3-, and 5-yr overall survival in GSE41613 cohort. HNSCC = head and neck squamous cell carcinoma, LRGs = lysosomes-related genes.

To develop a prognostic risk feature based on LRGs, we performed lasso and multivariate Cox regression analyses following univariate Cox regression analysis. Lasso Cox regression analysis reduced the initial 116 prognostic-related LRGs to 42 (Fig. 3A and B), while multivariate Cox regression ultimately included 13 independent prognostic LRGs for model construction, with the chromosomal locations of these genes displayed in Figure 3C. The risk score formula based on these 13 LRGs is as follows: riskscore = 0.5952084 * TPP1 + 0.8725288 * TOM1L1 - 0.6875402 * TMEM63A + 0.9366700 * TMEM192 + 0.9738867 * TM9SF1 + 0.6014700 * RRAGA + 2.3927221 * RNASE3 - 0.8357657 * HOOK3 - 1.8626369 * GPR137C + 0.2985203 * FTH1 - 0.7573946 * CYB561A3 - 0.6599438 * CTNS - 2.5215588 * ABCB6. This risk model can stratify TCGA-HNSCC and GSE41613 into high-risk and low-risk groups based on the median value (Fig. 3D–G). In the TCGA-HNSCC cohort, the high-risk group had a significantly worse prognosis than the low-risk group (Fig. 3H), and the AUC values for predicting 1, 3, and 5-year overall survival of HNSCC patients based on the risk score were 0.739, 0.08, and 0.806, respectively (Fig. 3I). Similarly, in the GSE41613 cohort, patients in the high-risk group had a worse prognosis than those in the low-risk group (Fig. 3J), and the AUC values for predicting 1, 3, and 5-year overall survival based on the risk score were 0.634, 0.608, and 0.597, respectively (Fig. 3K).

### 3.3. Clinical relevance of risk signature derived from LRGs

**Figure 4.**
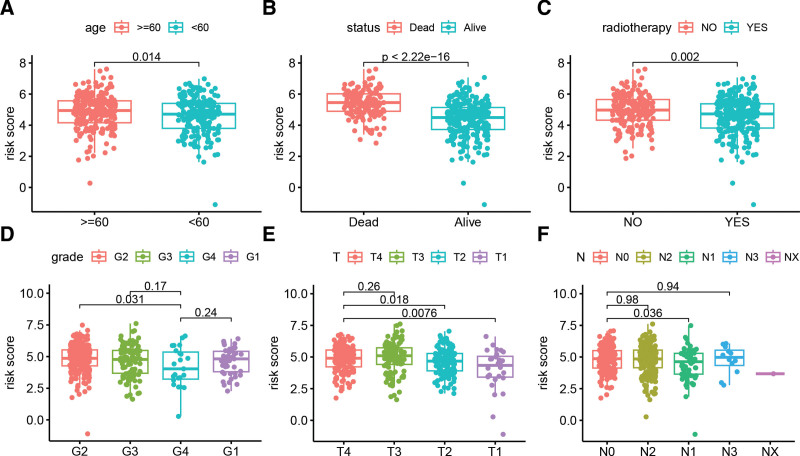
Relationship between LRG-derived HNSCC prognostic model and clinical pathological features. (A–F) Riskscore comparison among different age groups, survival outcomes, radiotherapy, grade, T stage, and N stage stratification. HNSCC = head and neck squamous cell carcinoma, LRGs = lysosomes-related genes.

We conducted a comprehensive analysis (Fig. 4A–F) to compare the differences in risk scores among various clinical and pathological feature groups. Our results revealed that elderly patients (>=60) had significantly higher riskscores than younger patients (<60) (*P* = .014). Patients who experienced death as an outcome had a higher riskscore compared to those who survived (*P* = 2.22e-16), and patients who did not receive radiotherapy had a better riskscore than those who did (*P* = .002). Furthermore, we observed that the risk score of G4 grade was significantly lower than that of G2 grade (*P* = .031), and the riskscore of patients with T4 stage was significantly higher than that of T2 (*P* = .018) and T1 stage (*P* = .0076), and the riskscore of patients with N0 stage was significantly higher than that of N1 stage (*P* = .036). These findings suggest that LRGs-derived risk signature are associated with age, survival outcome, radiotherapy, grade, T and N staging, and high-risk patients have higher clinical staging and adverse outcomes.

### 3.4. Pathways related to risk signature derived from LRGs

**Figure 5.**
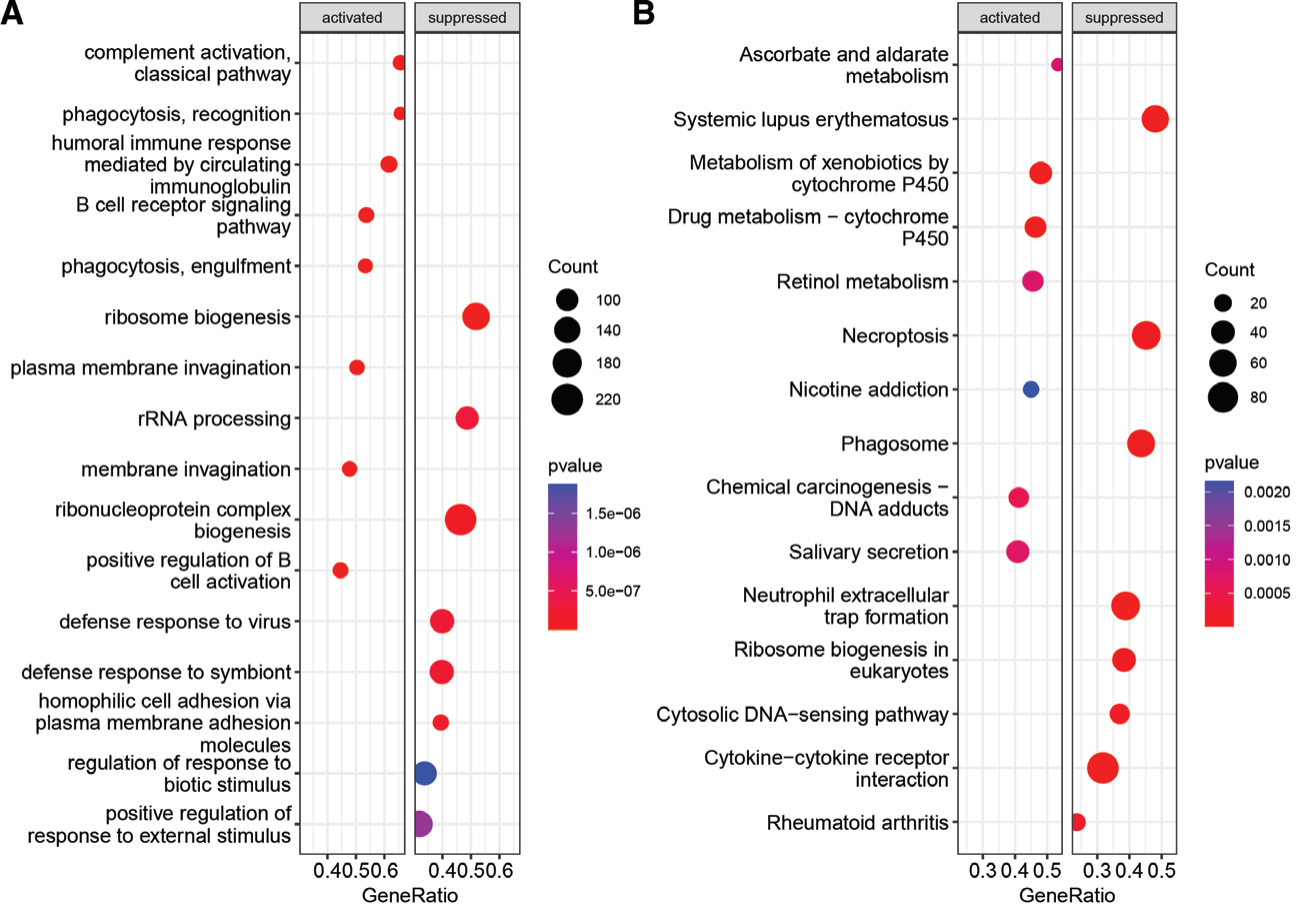
Gene expression GSEA analysis of high-risk and low-risk groups. (A) Gene ontology terms significantly activated and inhibited in the low-risk group compared to the high-risk group. (B) KEGG pathways significantly activated and inhibited in the low-risk group compared to the high-risk group.

To investigate the pathways and biological processes associated with the LRG-derived risk model, we performed gene set enrichment analysis. Our results indicated that immune-related processes were significantly activated in the low-risk group compared to the high-risk group, including positive regulation of B cell activation, humoral immune response, and B cell receptor signaling pathway. In contrast, processes such as ribosome biogenesis, ribonucleoprotein complex biogenesis, and rRNA processing were significantly suppressed (Fig. 5A). Additionally, KEGG enrichment analysis revealed that pathways such as metabolism of xenobiotics by cytochrome P450 and salivary secretion were significantly activated in the low-risk group, while pathways such as cytokine-cytokine receptor interaction, necroptosis, phagosome, and neutrophil extracellular trap formation were significantly suppressed (Fig. 5B).

### 3.5. Relationship between risk signature derived from LRGs and immune infiltration

**Figure 6.**
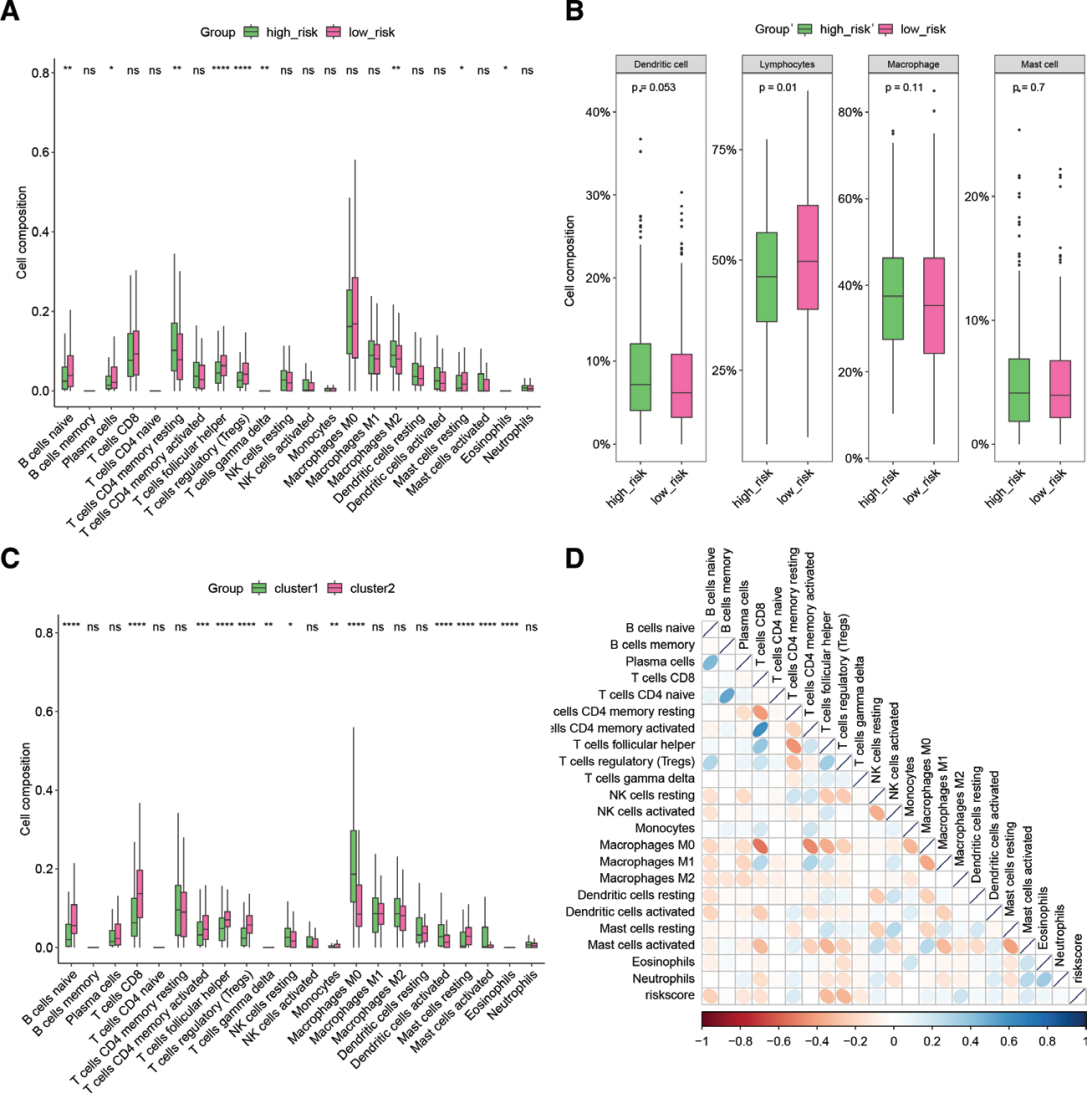
Relationship between LRG-derived risk model and molecular subtypes and immune infiltration. (A) Comparison of immune cell infiltration in tumor tissues of HNSCC patients in high-risk and low-risk groups. (B) Comparison of infiltration of 4 types of immune cells in high-risk and low-risk groups. (C) Comparison of immune cell infiltration among molecular subtypes derived from LRGs. (D) Correlation analysis between riskscore and infiltration of 22 immune cells. *P < .05, **P < .01, ***P < .001, ****P < .0001. HNSCC = head and neck squamous cell carcinoma, LRGs = lysosomes-related genes.

In order to evaluate the immune infiltration of HNSCC patients and its correlation with the LRG-derived risk model, we computed the 22 immune cell compositions in the tumor tissues of TCGA-HNSCC cohort patients. Our findings showed significant differences in 9 immune cells between the high-risk group and the low-risk group, including naive B cells, CD8 T cells, resting CD4 memory T cells, follicular helper T cells, regulatory T cells, and M2 macrophages (Fig. 6A). Upon categorization, lymphocytes were significantly lower in the high-risk group than in the low-risk group (Fig. 6B). Furthermore, significant differences in immune cell infiltration were observed among LRG-derived HNSCC molecular subtypes (Fig. 6C). From a prognostic perspective, the B cell and most T cell infiltration were generally consistent in groups with better prognosis, such as cluster 2 and the low-risk group. Additionally, correlation analysis revealed significant correlations between riskscore and most immune cell infiltrations (Fig. 6D).

### 3.6. Prediction of treatment response by risk signature derived from LRGs

**Figure 7.**
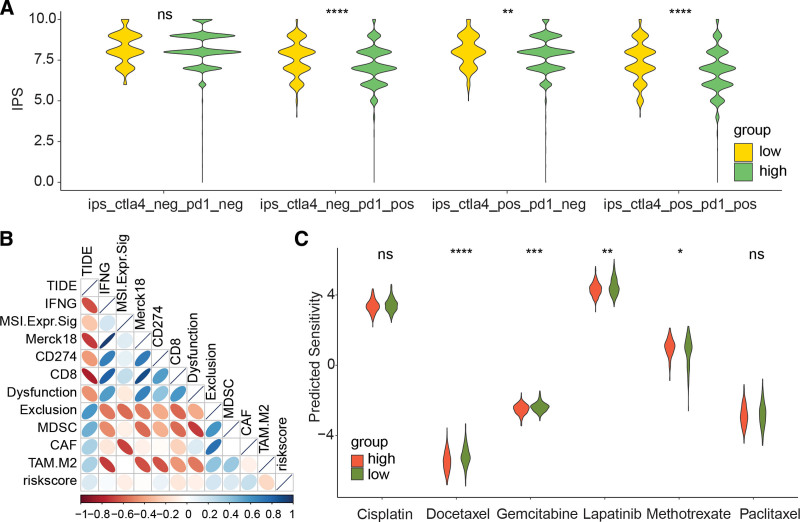
Relationship between LRG-derived risk model and treatment response. (A) Comparison of IPS scores in high-risk and low-risk groups. (B) Correlation between riskscore and TIDE algorithm score. (C) Comparison of sensitivity to 6 chemotherapy drugs in high-risk and low-risk groups. *P < .05, **P < .01, ***P < .001, ****P < .0001. IPS = immunophenoscore, LRGs = lysosomes-related genes.

Not all patients respond to immunotherapy, therefore, we evaluated the response of high-risk and low-risk patients to immunotherapy. Our findings, as depicted in Figure 7A, indicate that low-risk patients have a higher IPS in CTLA-4 negative PD1 positive, CTLA-4 positive PD1 positive, and CTLA-4 positive PD1 negative situations. This suggests a higher likelihood of response to immunotherapy and benefit from it. Additionally, our analysis revealed that riskscore is significantly positively correlated with TIDE, cancer-associated fibroblasts, Exclusion, and other factors (Fig. 7B), further supporting the notion that low-risk patients have a better response to immunotherapy. Furthermore, our chemotherapy sensitivity analysis showed that high-risk patients are more sensitive to docetaxel, gemcitabine, lapatinib, and methotrexate compared to low-risk patients (Fig. 7C). This indicates a higher likelihood of benefit from these chemotherapy drugs.

### 3.7. Construction of nomogram to evaluate HNSCC prognosis

**Figure 8.**
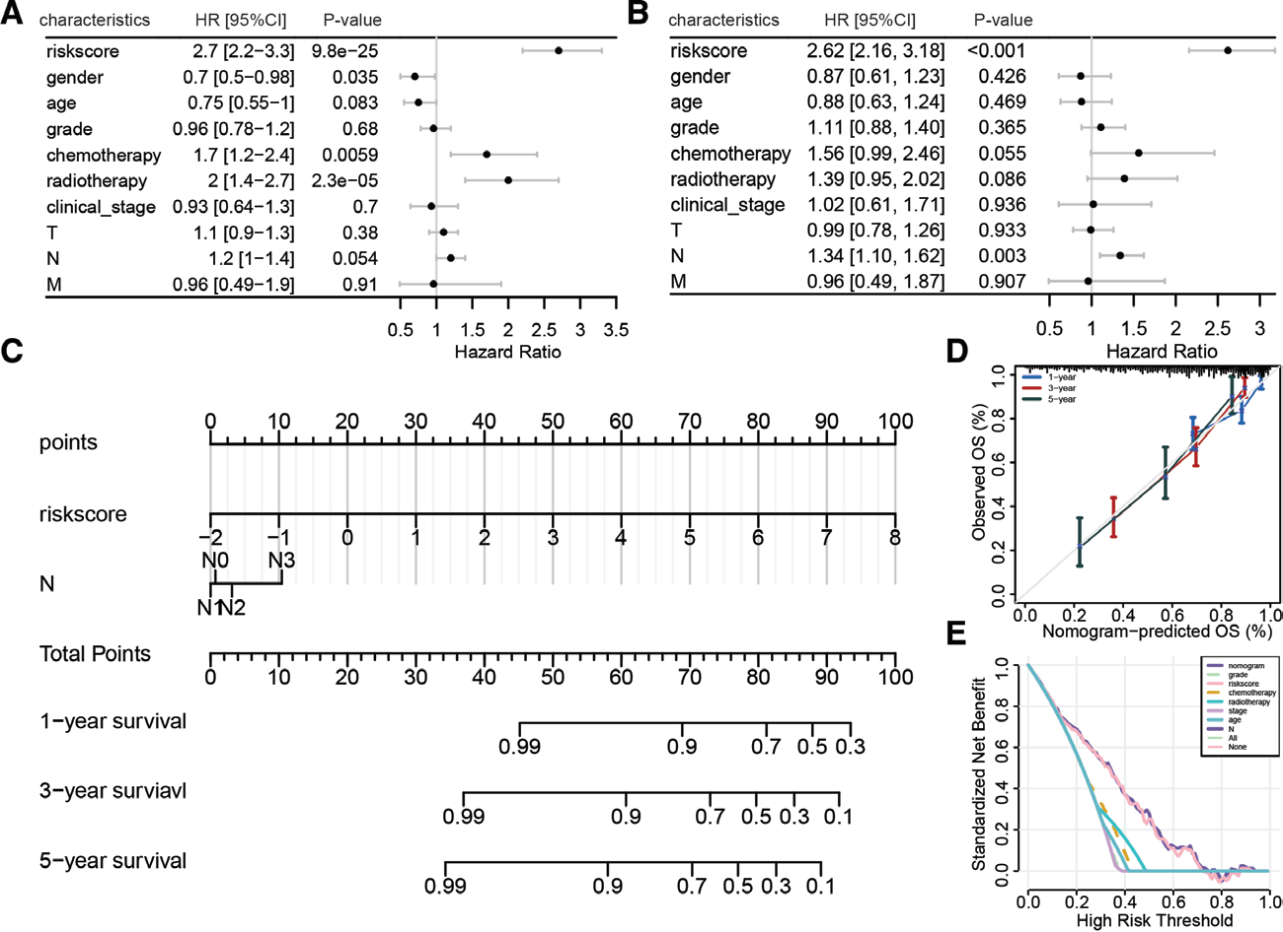
Construction of nomogram model for HNSCC prognostic evaluation. (A) Forest plot of univariate Cox regression analysis of riskscore and other clinical pathological features. (B) Forest plot of multivariate Cox regression analysis of riskscore and other clinical pathological features. (C) Nomogram composed of riskscore and N stage. (D) Calibration curve of nomogram model in predicting 1, 3, and 5-yr overall survival in HNSCC patients. (E) Decision curve analysis of nomogram model and other prognostic factors for predicting overall survival. HNSCC = head and neck squamous cell carcinoma.

To further investigate the clinical applicability of the risk model derived from LRGs, we developed a nomogram to predict the 1, 3, and 5-year overall survival of HNSCC patients. Initially, we identified riskscore, gender, chemotherapy, and radiotherapy as prognostic factors for HNSCC (Fig. 8A). Subsequently, multivariate Cox regression analysis revealed that riskscore and N stage were 2 independent prognostic factors for HNSCC (Fig. 8B). As a result, we constructed a nomogram that integrated risk features derived from LRGs and N stage (Fig. 8C). The calibration curve demonstrated that the predicted results of this nomogram for the 1, 3, and 5-year overall survival of HNSCC patients were highly consistent with the actual situation (Fig. 8D), validating its effectiveness in predicting HNSCC prognosis. Additionally, the decision curve analysis showed that compared to other prognostic factors, this nomogram had a superior net benefit in predicting the 1-year overall survival of HNSCC patients (Fig. 8E).

## 4. Discussion

Lysosome-related genes (LRGs) have shown promise as prognostic indicators for HNSCC. For instance, late endosomal/lysosomal adaptor and MAPK and mTOR activator 5 has been shown to promote cell proliferation, migration, and angiogenesis, and its overexpression in HNSCC is associated with poor prognosis.^[15]^ Similarly, overexpression of lysosomal-associated transmembrane protein in HNSCC is associated with tumor staging and lymph node metastasis, making it a poor prognostic indicator for this disease.^[14]^ In our study, we systematically evaluated the prognostic value of LRGs in HNSCC and identified 116 LRGs significantly associated with HNSCC prognosis. Based on these LRGs, we identified 2 subtypes with significant differences in prognosis and immune infiltration. Furthermore, the risk features based on 13 LRGs showed great potential for predicting HNSCC prognosis with excellent performance. These findings suggest that LRGs may serve as promising prognostic biomarkers and therapeutic targets for managing HNSCC.

The genes identified in this risk feature have been implicated in cancer or HNSCC pathogenesis. For example, TPP1 is a telomere-binding protein that is a crucial component of the mammalian telomere protection protein complex. Increased expression of TPP1 in colorectal cancer cells can protect telomeres from DNA damage and confer radiation resistance.^[16]^ Target of myb1 like 1 membrane trafficking protein has both Golgi transport and signaling functions, and can drive membrane delivery of membrane-Type 1 matrix metalloproteinase, promoting ErbB2-induced breast cancer cell invasion.^[17]^ Transmembrane protein 63A gene knockout can inhibit diffuse large B-cell lymphoma cell proliferation and is a potential therapeutic target.^[18]^ Transmembrane 9 superfamily member 1 is a target of phosphorylated CTD interacting factor 1 and may mediate phosphorylated CTD interacting factor 1 oncogenic function.^[19]^ Hook microtubule tethering protein 3 is an independent prognostic factor for prostate cancer^[20]^ and is associated with cisplatin resistance in lung cancer.^[21]^ Ferritin heavy chain 1 (FTH1) plays a significant anti-growth role in breast cancer cells by inhibiting c-MYC expression,^[22]^ and FTH1-based iron death regulation plays an important role in cancer.^[23]^ Cytochrome b561 family member A3 is an iron reductase, and its gene knockout leads to catastrophic lysosomal and mitochondrial damage.^[24]^ ATP binding cassette subfamily B member 6 is an ABC transporter, and its mRNA and DNA methylation levels are associated with early intrahepatic recurrence of hepatitis C virus-related liver cancer.^[25]^ Although these studies suggest the possible prognostic potential and biological functions of these genes in HNSCC, further validation is required.

This study highlights differences in immune cell infiltration among different molecular subtypes and risk groups of HNSCC, indicating the potential involvement of B cells, T cells, macrophages, and mast cells in HNSCC prognosis. B cell infiltration was found to increase in subtypes and risk groups with better prognosis, primarily involved in humoral immunity. B cells secrete immune regulatory factors, such as cytokines and antibodies, which play an important role in immune surveillance and clearance of tumors.^[26]^ Follicular helper T cells and regulatory T cells are associated with good prognosis in HNSCC and are significantly enriched in the low-risk group. CD4 Follicular helper T cell infiltration is associated with breast cancer prognosis,^[27]^ and it can synergistically promote anti-tumor CD8 T cell response.^[28]^ In addition, Follicular helper T cells also mediate the response of breast cancer mouse models to checkpoint inhibitors.^[29]^ Regulatory T cells are an immunosuppressive subset of CD4 + T cells, and Treg cells maintain immune homeostasis by preventing excessive immune activation. However, Treg infiltration can accelerate tumor immune escape, impair the host anti-tumor immune response, and promote tumor progression. This finding contradicts our study results; therefore, further exploration is needed to understand the role and mechanism of Treg infiltration in HNSCC.

Immunotherapy is a promising cancer treatment modality, but its efficacy varies among patients. Therefore, it is essential to evaluate the immune therapy response of different patients and develop individualized treatment plans to improve patient prognosis. In this study, we demonstrated the potential value of evaluating immune therapy response based on risk features of LRGs, which may be associated with immune cell infiltration.^[30]^ A comprehensive analysis of tumor-infiltrating immune cells can reveal the mechanisms of cancer immune evasion and provide opportunities for developing new treatment strategies. Furthermore, our findings suggest that high-risk and low-risk patients exhibit distinct sensitivities to certain chemotherapy drugs, indicating that these model genes may be potential targets for drug resistance. Hence, further exploration into the roles and mechanisms of these genes in HNSCC drug response is warranted.

Our study developed a novel HNSCC prognostic risk model, which can assist clinicians in predicting the likelihood of disease recurrence or progression, and provide information for treatment decision-making. However, this study has some limitations that need to be addressed. Firstly, there remains a need for full validation of molecular subtyping and model construction based on LRGs, with additional work required to develop their clinical applicability. Secondly, the biological functions of key LRGs have yet to be fully explored and elucidated, necessitating further experimentation to better understand their roles in HNSCC.

## 5. Conclusion

In summary, this study identified 116 prognostic-related LRGs in HNSCC and identified 2 molecular subtypes with significant heterogeneity based on these LRGs. A risk feature consisting of 13 LRGs was constructed, which can be applied to HNSCC prognosis prediction, immune therapy response, and drug sensitivity evaluation. However, these molecular subtypes and risk models need further clinical validation.

## Author contributions

**Conceptualization:** Aichun Zhang.

**Data curation:** Yangzi Jin.

**Formal analysis:** Aichun Zhang, Yangzi Jin, Xinbo Zou.

**Funding acquisition:** Aichun Zhang.

**Investigation:** Aichun Zhang.

**Methodology:** Aichun Zhang.

**Project administration:** Aichun Zhang.

**Resources:** Yangzi Jin.

**Software:** Yangzi Jin.

**Supervision:** Aichun Zhang, Shou Zhang.

**Validation:** Aichun Zhang, Yangzi Jin.

**Visualization:** Aichun Zhang, Xinbo Zou.

**Writing – original draft:** Aichun Zhang.

**Writing – review & editing:** Shou Zhang.

## Supplementary Material


